# Bibliometric analysis of Crohn's disease in children, 2014–2024

**DOI:** 10.3389/fped.2025.1515251

**Published:** 2025-03-06

**Authors:** Yanjing Zhu, Yunhong Ma, Zhengjiu Cui, Yueli Pan, Juanjuan Diao

**Affiliations:** ^1^ The First Clinical Medical College, Shandong University of Traditional Chinese Medicine, Jinan, China; ^2^ Department of Pediatrics, Affiliated Hospital of Shandong University of Traditional Chinese Medicine, Jinan, China; ^3^ Department of Pediatrics, Haining Hospital of Traditional Chinese Medicine, Jiaxing, China; ^4^ Department of Pediatrics, Affiliated Hospital of Nanjing University of Chinese Medicine, Nanjing, China

**Keywords:** childhood Crohn's disease, bibliometric analysis, visual analysis, VOSviewer, citespace

## Abstract

**Background:**

In recent years, the incidence of Crohn's disease has risen significantly in the pediatric population, and its prolongation has had a major impact on children's physical and mental health as well as on the quality of life of their families, and has therefore received extensive attention from scholars around the world. A large number of articles have been published on Crohn's disease in children; however, there is still a lack of studies that use visualization methods for bibliometric analysis of relevant literature. The purpose of this paper is to statistically analyze the published literature in the field of Crohn's disease in children in order to help in the future diagnosis and treatment of Crohn's disease in children.

**Methods:**

Literature meeting the inclusion criteria was screened from the Web of Science Core Collection database. Literature was visualized and analyzed by author, country, institution, journal, reference, and keyword using Citespace (6.2.R4) and VOSviewer (1.6.18).

**Results:**

A total of 3,177 eligible publications were included. There is an overall increasing trend in the number of annual publications.Turner, Dan published the most number of articles with 78 and has a high impact in the field of CD. The most cited author was Levine, A. Among countries and institutions, the United States and Tel Aviv University had the highest number of publications. The journal with the most publications is Journal Of Pediatric Gastroenterology And Nutrition. The most co-cited journal was Inflammatory Bowel Diseases. The most cited document was ESPGHAN Revised Porto Criteria for the Diagnosis of Inflammatory Bowel Disease in Children and Adolescents, and the most cited document with the highest outbreak intensity was The Medical Management of Paediatric Crohn's Disease: an ECCO-ESPGHAN Guideline Update.The most frequent keyword was “inflammatory bowel disease”.

**Conclusion:**

This study provides a visual summary of information for the field of pediatric Crohn's disease and contributes to international collaboration to promote research in the field of pediatric Crohn's disease.

## 1 Preamble

Crohn's disease (CD) is a chronic non-specific inflammatory disease of the digestive tract, which was first reported in detail by Crohn in 1932 as “regional ileitis”, and was later formally named Crohn's disease by the medical community in honor of Dr. Crohn. Crohn's disease was first reported in detail by Crohn in 1932. The incidence of the disease has been on the rise globally and has gradually evolved into a global disease, especially in countries where the incidence of the disease was relatively low in the past (1, 2). Crohn's disease has received a lot of attention from scholars around the world, especially in countries where the incidence was previously low. Crohn's disease has no fixed age group, but up to a quarter of Crohn's patients are children (3). The etiology of Crohn's disease (CD) in children is unclear and may be related to environmental factors, genetic factors, immunologic factors, infections, and intestinal flora disorders. Clinical manifestations are characterized by chronic, recurrent gastrointestinal symptoms such as abdominal pain, diarrhea, and blood in the stool, as well as extra-intestinal symptoms such as malnutrition, short stature, developmental delays, and suboptimal bone health (4–6). Due to its seriousness and prolonged course, it seriously affects children's health and quality of life. Children and adolescents with Crohn's disease (CD) tend to have a more complex course than adult patients, and this chronic inflammatory disease not only has a greater impact on the physical health of the children themselves, but also poses a significant challenge to their families in terms of mental health and long-term therapeutic management. A large number of articles have been published on Children and adolescents with Crohn's disease (CD). However, there is a lack of studies that use visualization methods for bibliometric analysis of relevant literature. The aim of this study was to characterize the literature output on Crohn's disease comprehensively between 2014 and 2024 through bibliometric analysis, to identify the hotspots and frontiers of research, and to provide new ideas and references for future clinical and research work.

Bibliometrics refers to the use of mathematical and statistical methods to mine the information of a subject area, after we sort out and summarize the information we get, we use visual data and charts to express the history of research, research status, research hotspots and development trends of the subject, so that the researchers can have a more direct understanding of the results of the research of the subject area.Citespace and VOSviewer are both software tools to build and visualize the network of bibliometrics. Citespace and VOSviewer are both software tools for building and visualizing bibliometric networks; Citespace generates a “scientific knowledge graph” through econometric analyses of literature in a specific field, helping us to understand the past research trajectory and future research prospects of the subject area (7–9). Vosviewer can be constructed based on citations, co-occurrences, co-authors, bibliographic couplings, and collaborations, and can also construct and visualize co-occurrence networks of important terms extracted from the vast scientific literature (10, 11). It can also build and visualize co-occurrence networks of important terms extracted from large amounts of scientific literature. In recent years, bibliometrics and visual analytics have been widely used in the field of medicine.The Web of Science Core Collection (WOS) is the world's largest comprehensive multidisciplinary core journal database. To the best of our knowledge, no bibliometric analysis of Crohn's disease in children has been reported through the above authoritative databases.

In this study, we used Citespace (6.2.R4) and VOSviewer (1.6.18) to statistically analyze and visualize literature related to childhood CD in the WOS Core Collection database from 2014 to 2024. Our ultimate goals were mainly (i) to identify influential countries, institutions, authors and journals that have made outstanding contributions to the field of pediatric CD; (ii) to shed light on the current status, hotspots and future trends of research; and (iii) to promote collaborative research among scholars globally in the hotspot and bottleneck areas of pediatric CD.

## 2 Materials and methods

### 2.1 Data sources

**Figure 1:**
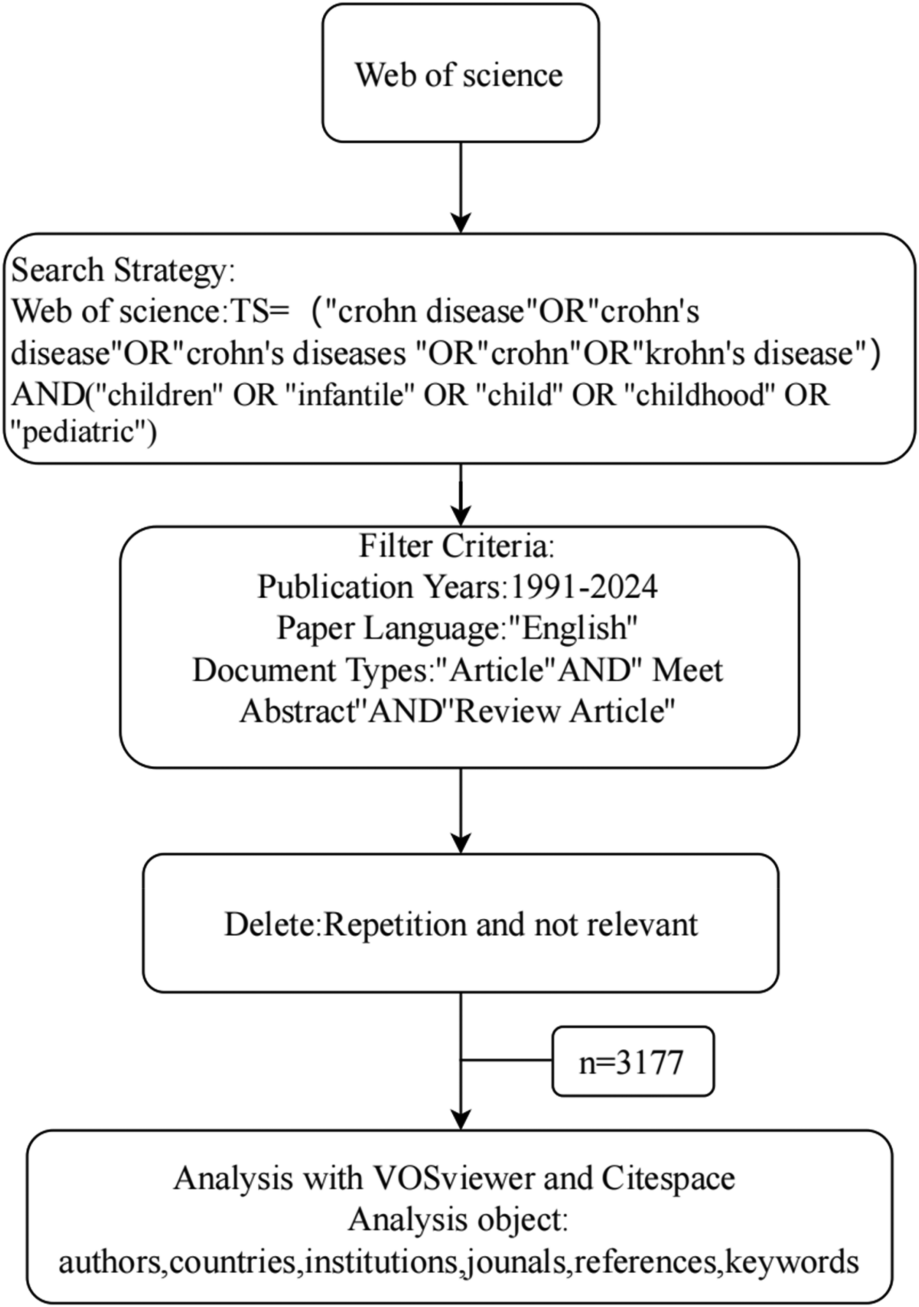
Flowchart of literature collection and selection.

The literature was searched on August 2, 2024 from the WOS database. The following search terms were used in the subject: (“crohn disease” OR “crohn's disease” OR “crohn's diseases” OR “crohn” OR “krohn's disease”) AND (“children” OR “infantile” OR “child” OR “childhood” OR “pediatric”), and the search timeframe was limited to January 1, 2014 to August 2, 2024. The article types selected were articles and review papers. The language selected was “English”. 3,177 articles were collected from the eligible databases. Two authors (Yanjing Zhu and Yunhong Ma) collected all the data independently, and the literature information of the databases were exported in txt format. Cui Zhengjiu and Pan Yueli deleted duplicates and irrelevant documents. Finally, a total of 3,177 documents were included in this study, and the specific search process is shown in Figure 1.

### 2.2 Data analysis

We used Excel to count the annual number of publications, countries of publication, publishing organizations, and authors of publications. We combined Taiwan, Hong Kong, Macau, and the People's Republic of China into China (CHINA). We also used Citespace (6.2.R4) and VOSviewer (1.6.18) to extract basic noun phrases from the titles, abstracts, and keywords of the literature for literature co-occurrence analysis. We used both software for mapping to capture important contributors and research hotspots in pediatric CD. In Citespace, the time span was selected as 2014–2024, the time node was set to 1 year, the node type was selected as country, keyword, etc., the node intensity was defaulted to cosine, and the threshold was selected as K = 5. In the analysis of keywords over time, VOSviewer was used to analyze the countries, institutions, authors, journals, and keywords. The data type is selected as WOS, the counting method is set as complete counting, and then the minimum frequency of occurrence of words is set to finalize the graph.

## 3 Results-based

### 3.1 Trends in the number of publications

**Figure 2:**
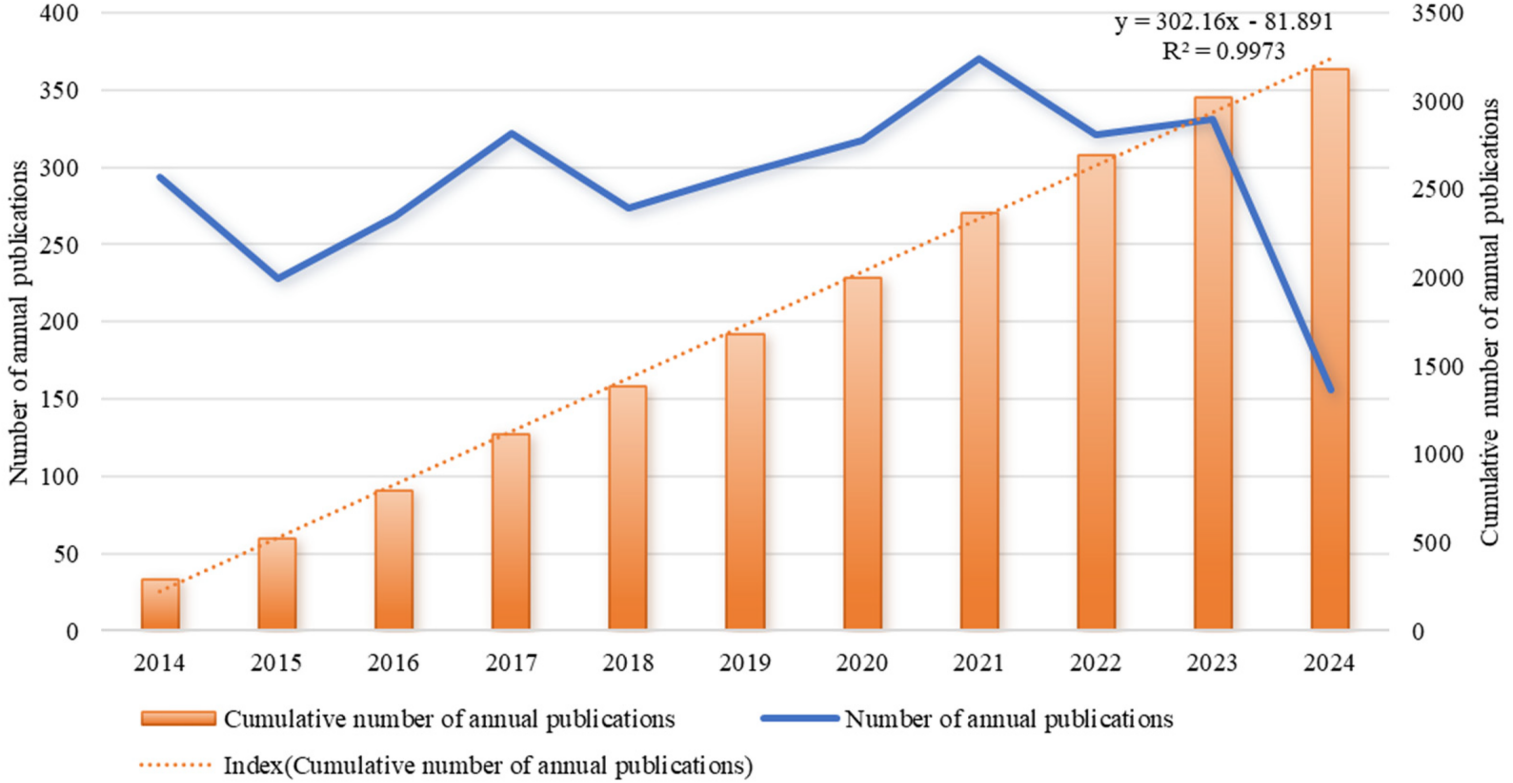
Trend of publications in the field of children's CD (2014–2024).

As shown in Figure 2, which depicts the specific number and annual trend of CD-related publications from 2014 to 2024, there is an overall steady growth trend. The fastest growth rate was observed in 2017, a period of booming prosperity, with related publications peaking in 2021 (*n* = 370). Since 2020, CD-related publications are all above 300, and as of the retrieval date, the number of publications in 2024 is only 156, but combining with the trend of publications in recent years, the author predicts that this year's publications can still reach more than 300. From the orange trend line, it can be seen that the annual cumulative number of publications conforms to linear growth. The correlation coefficient *R*^2^ between the annual cumulative number of articles and the year of publication is 0.9973, indicating a strong correlation between the annual cumulative number of articles and the year. The above data show that in recent years, the field of pediatric CD has attracted more and more attention from scientists around the world, especially after 2020, when the field has become a hot topic with fruitful research results.

### 3.2 Authors and cited authors

**Figure 3:**
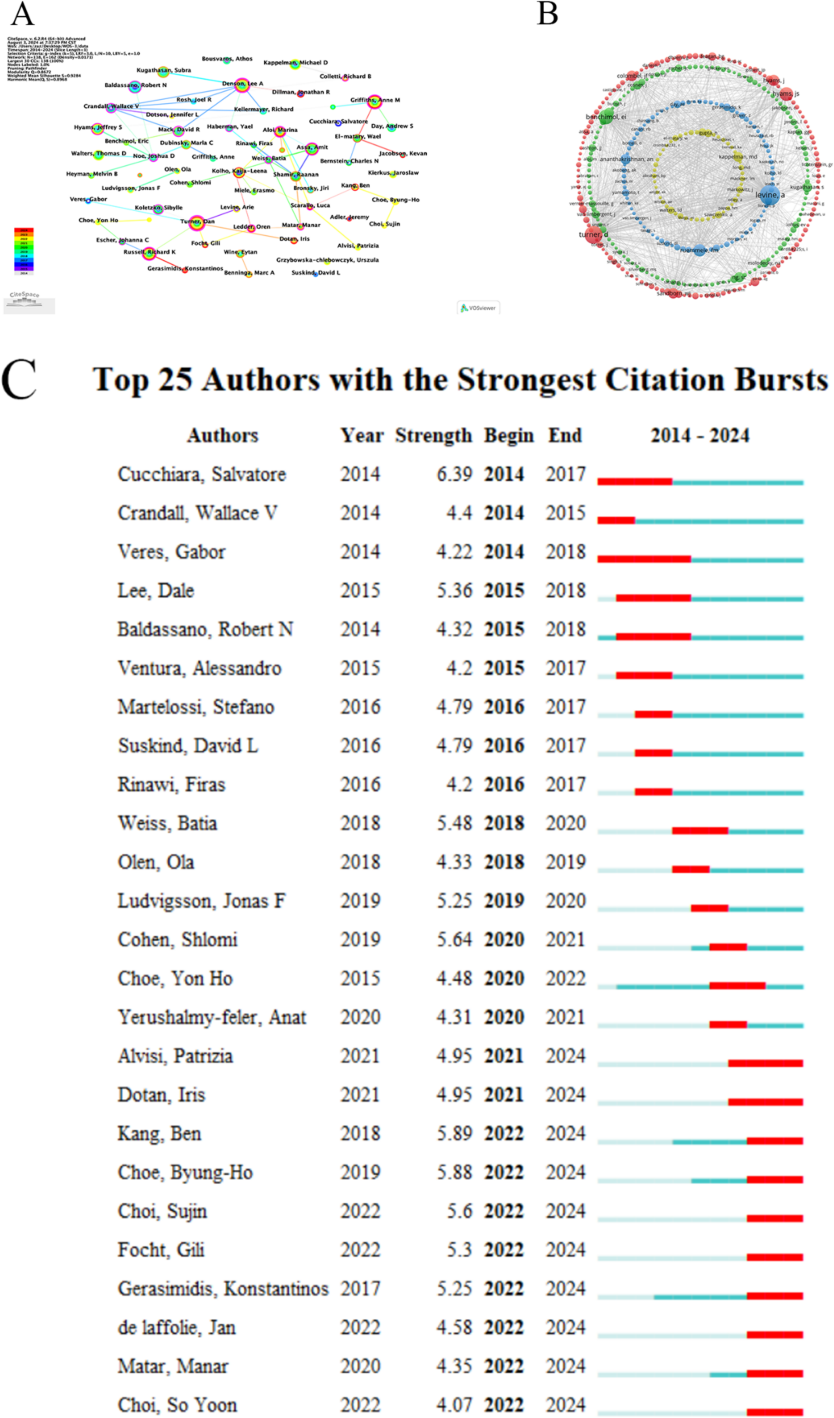
Analyse of authors and co-cited authors. **(A)** Visualization of Authors. **(B)** Visualization of Cited Authors. **(C)** The top 10 Cited Authors with the Strongest Citation Bursts.

The analysis of authors as well as the most cited authors in the references helps to understand the collaborative relationship between authors and to strengthen their collaboration and to identify the main contributors to the CD field. From the perspective of author analysis, the top 10 most prolific authors in the last 10 years are ranked as shown in Table 1. Turner, Dan was the most prolific author with a total of 79 publications. He is closely followed by Denson, Lee A who has published a total of 69 articles.Griffiths, Anne M ranks third with a total of 68 articles. The authors with the most citations in the references of their articles, whose citation counts can be used as a key indicator of the authors' contributions, are shown in Table 1, which shows the top 10 authors with the most citations in their references, all of whom have more than 300 citations. Levine, A is the most cited author, followed by Turner, D; Benchimol, Ei, Hyams, Js in top of the list in terms of number of publications and citations, which is a good indication that the authors have made outstanding contributions to the field. The author and co-cited author collaboration network visualization is performed by Citespace and VOSviewer, as shown in Figure 3A, where the node size reflects the frequency of authors' appearances, the connecting line between nodes indicates the co-occurrence relationship, its thickness indicates the intensity of co-occurrence, and the color corresponds to the time of the node's first co-occurrence. The cited authors are visualized as shown in Figure 3B, where each color represents a collaborative network and the node size indicates the magnitude of the frequency, from which it can be seen that among the top 10 cited authors, Levine, A; Turner, D; and Benchimol, Ei dominate their respective clusters. As can be seen in Figure 3C, among the strongest citation bursts cited by the top 10 authors from 2014 to 2024, Cucchiara,Salvatore has the highest intensity and Veres,Gabor has the longest duration.

**Table 1:** Top 10 authors of the number of publications and most cited authors.

Rank	Authors	Documents	Authors	Cications
1	Turner, Dan	78	Levine, A	1,561
2	Denson, Lee A	69	Turner, D	1,210
3	Griffiths, Anne M	68	Benchimol, Ei	801
4	Russell, Richard K	59	Hyams, Js	704
5	Assa, Amit	54	Ruemmele, Fm	573
6	Kugathasan, Subra	53	Hyams, J	457
7	Kolho, Kaija-Leena	52	Sandborn, Wj	423
8	Hyams, Jeffrey S	49	Ananthakrishnan, An	414
9	Baldassano, Robert N	44	Colombel, Jf	413
10	Kappelman, Michael D	43	Ng, SC	391

### 3.3 Countries and institutions

**Figure 4:**
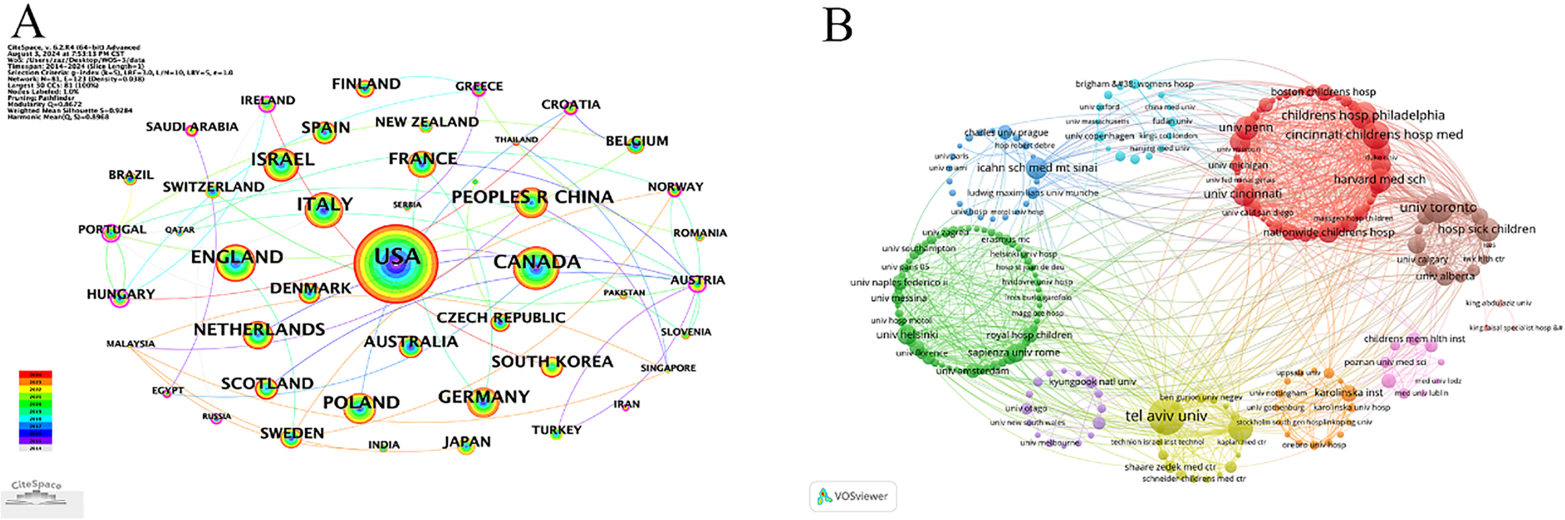
Analysis of the countries and institutions, **(A)** visualization of countries,each node represents a country, the size of the node indicates the publication output of the country, and high centrality (<0.1) is represented by nodes with purple rings. (<0.1) is represented by nodes with purple rings. **(B)** Visualization of institutions.

The visual analysis of countries and institutions was performed by Citespace and VOSviewer, and as can be seen in Table 2, the United States has the highest number of publications with 451 articles, followed by Canada with 190 and the United Kingdom with 174, and Germany and Italy are also at a high level, suggesting that there is a high level of interest in research in this area in these countries. Combined with Figure 4A shows that Hungary, has the highest centrality and the nodes are shown as purple rings, suggesting that this country is working more closely with other countries in this area of CD in children, and acts as a bridge in national exchanges. The top 10 institutions in terms of number of publications and their countries are given in Table 3.Tel Aviv University (159) leads in terms of number of publications, followed by University of Toronto (122) and Cincinnati Children's Hospital Medical (107). Six of the top 10 publishing institutions are located in the United States, but the top 5 publishers are from Israel, Canada, and the United States, suggesting that Israel and Canada are not weaker than the United States in this area of research. Figure 4B visualizes the co-occurrence network between institutions, and in combination with Table 3, it can be seen that the institutions with a high number of publications are located in the red cluster, which suggests that the collaborative network of the red cluster has a high impact in the field of children's CD. Cincinnati Children's Hospital Medical and Childrens hospital philadelphia are closely related in the red cluster; The Hospital for Sick Children and The Hospital for Sick Children collaborate more and work closely with organizations in other clusters.

**Table 2:** Top 10 countries of the number of publications.

Rank	Countries	Count
1	USA	451
2	Canada	190
3	Italy	174
4	England	76
5	Israel	70
6	China	67
7	Poland	55
8	Germany	52
9	Netherlands	48
10	France	46

**Table 3:** Top 10 institutions by number of publications.

Rank	Institutions	Count	Original country
1	Tel Aviv University	**159**	Israel
2	University of Toronto	**122**	Canada
3	Cincinnati Children's Hospital Medical	**107**	USA
4	Childrens hospital philadelphia	**104**	USA
5	The Hebrew University of Jerusalem	**88**	Israel
6	The Hospital for Sick Children	**87**	Canada
7	University of Pennsylvania	**84**	USA
8	Harvard Medical School	**84**	USA
9	University of Cincinnati	**81**	USA
10	Icahn School of Medicine at Mount Sinai	**74**	USA

### 3.4 Periodicals

**Figure 5:**
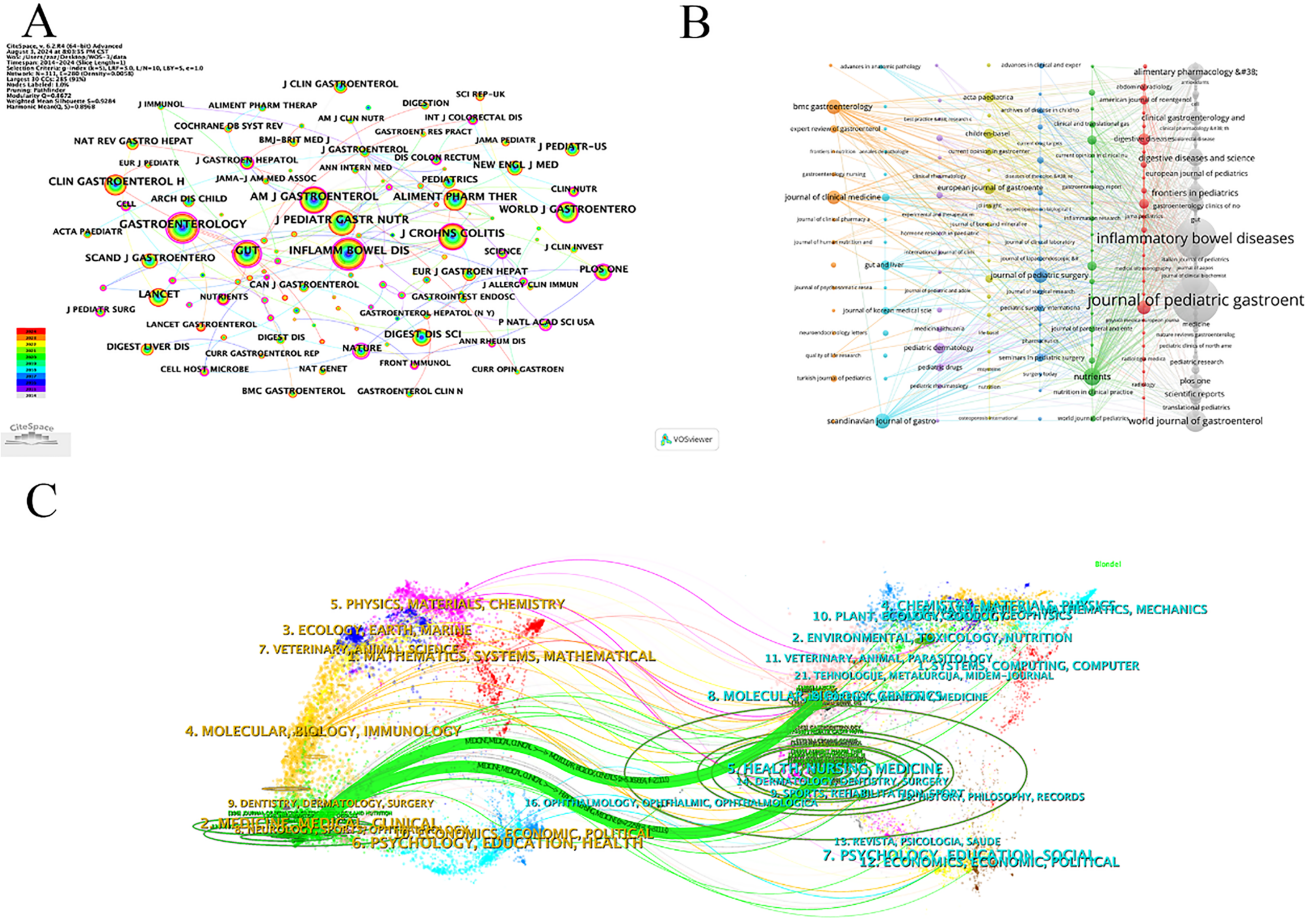
Analysis of the journals. **(A)** Visualization of journals. **(B)** Visualization of cited journals. **(C)** The dual-map overlay of LN in Children research.

As shown in Table 4, the most prolific journal was Journal Of Pediatric Gastroenterology And Nutrition (IF 2.4) with 396 publications, followed by Inflammatory Bowel Diseases (IF 4.5) with 304 publications. Inflammatory bowel diseases was categorized as Q1, indicating that this journal has a high academic reputation. In addition, Inflammatory Bowel Diseases was the most cited, followed by Gastroenterology (IF 25.7). From Table 4 and Table 5, it can be seen that most of the journals are Q1 whether they are the most productive or the most cited journals, indicating that the research on pediatric CD is still of high significance and needs to be improved continuously. Figures 5A,B denote the citing and cited literature, respectively, and it can be seen from the graphs that the largest nodes of the two are the Journal Of Pediatric Gastroenterology And Nutrition, Inflammatory bowel disease, and Inflammatory Bowel Diseases ranked second in Figure 5A node size, indicating that the journal has greater influence and authority in the field of pediatric CD compared with other journals. This indicates that the journal has more influence and authority in the field of pediatric CD than other journals. The two-plot overlay of journals (Figure 4C) suggests the relationship between citing and cited journals, with the green line indicating the number of citations from journals in the field of Medicine/Medical/Clinical/Neurology/Sports/Ophthalmology/Dentistry/Dermatology/Surgery. Surgery domains cited journals mostly from the Health/Nursing/Medicine/Dermatology/Dentistry/Surgery/Sports/Rehabilitation Sport domains.

**Table 4:** Top 10 journals distributed by publications.

Rank	Journal	Publications	IF (JCR2023)	JCR quartile
1	Journal Of Pediatric Gastroenterology And Nutrition	396	2.4	Q3
2	Inflammatory Bowel Diseases	304	4.5	Q1
3	Journal Of Crohns & Colitis	134	8.3	Q1
4	World Journal Of Gastroenterology	86	4.3	Q1
5	Nutrients	55	4.8	Q1
6	Frontiers In Pediatrics	54	2.1	Q2
7	Alimentary Pharmacology & Therapeutics	50	6.6	Q1
8	Digestive Diseases And Sciences	46	2.5	Q2
9	Gastroenterology	46	25.7	Q1
10	Scandinavian Journal Of Gastroenterology	40	1.6	Q3

**Table 5:** Top 10 journals distributed by citations.

Rank	Cited journal	Total citations	IF (JCR2023)	JCR quartile
1	Inflammatory Bowel Diseases	2,716	4.5	Q1
2	Gastroenterology	2,391	25.7	Q1
3	Journal Of Pediatric Gastroenterology And Nutrition	2,199	24	Q3
4	Journal Of Crohns & Colitis	2,049	8.3	Q1
5	American Journal Of Gastroenterology	2,020	8	Q1
6	Gut	1,982	23	Q1
7	Alimentary Pharmacology & Therapeutics	1,576	6.6	Q1
8	Clinical Gastroenterology And Hepatology	1,465	11.6	Q1
9	World Journal Of Gastroenterology	1,334	4.3	Q1
10	Lancet	1,158	98.4	Q1

### 3.5 Analysis of references

**Figure 6:**
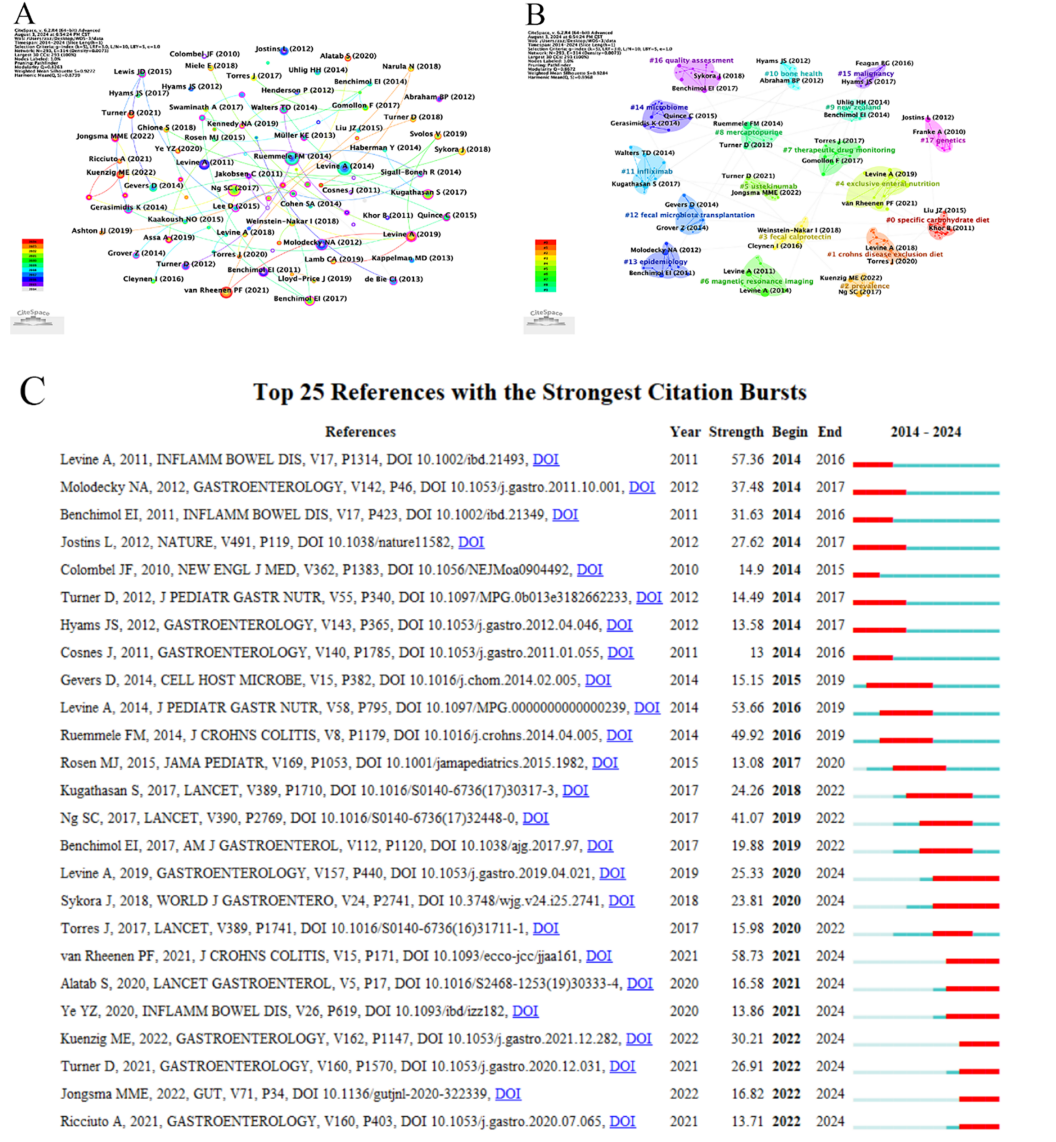
Analyse of co-cited references and references burst. **(A)** Visualization of Co-cited References. **(B)** Clustering map of reference co-citation related to research of crohn's disease. **(C)** The top 25 References with the Strongest Citation Bursts.

Literature co-citations were analyzed in conjunction with Table 6 and Figure 6. As shown in Table 6 and Figure 6A, the most cited literature was Levine A's article published in the Journal of Pediatric Gastroenterology and Nutrition in 2014 with 227 citations. The second most frequently cited article was published in the Journal of Crohns & Colitis in 2014.Combined with Figure 6C, it can be seen that the highest intensity of outbreaks was seen in Van Rheenen PF's article published in the Journal of Crohns & Colitis in 2021, which had a significant impact on the 2014 article published in this journal which updated the consensus guidelines on Children and adolescents with CD.Citespace provides cluster analysis of the co-cited literature, allowing us to visualize trends in literature co-citation and hot research topics. Therefore, Citespace was used for cluster analysis of the literature with Modularity Q of 0.8672. 18 clusters with a weighted Mean Silhouette of 0.9284 were clustered, and the literature in the same cluster was homogeneous, as shown in Figure 6A, and the clusters mainly covered the aspects of diagnosis, treatment, and management.

**Table 6:** Top 10 cited references with the highest citations of occurrence.

Rank	Cited reference	Citations
1	Levine A, 2014, J PEDIATR GASTR NUTR, V58, P795	227
2	Ruemmele FM, 2014, J CROHNS COLITIS, V8, P1179	197
3	Van Rheenen PF, 2021, J CROHNS COLITIS, V15, P171	171
4	Levine A, 2011, INFLAMM BOWEL DIS, V17, P1314	135
5	Ng SC, 2017, LANCET, V390, P2769	132
6	Molodecky NA, 2012, GASTROENTEROLOGY, V142, P46	123
7	Kugathasan S, 2017, LANCET, V389, P1710	104
8	Levine A, 2019, GASTROENTEROLOGY, V157, P440	102
9	Jostins L, 2012, NATURE, V491, P119	91
10	Benchimol EI, 2017, AM J GASTROENTEROL, V112, P1120	79

### 3.6 Keyword analysis

**Figure 7:**
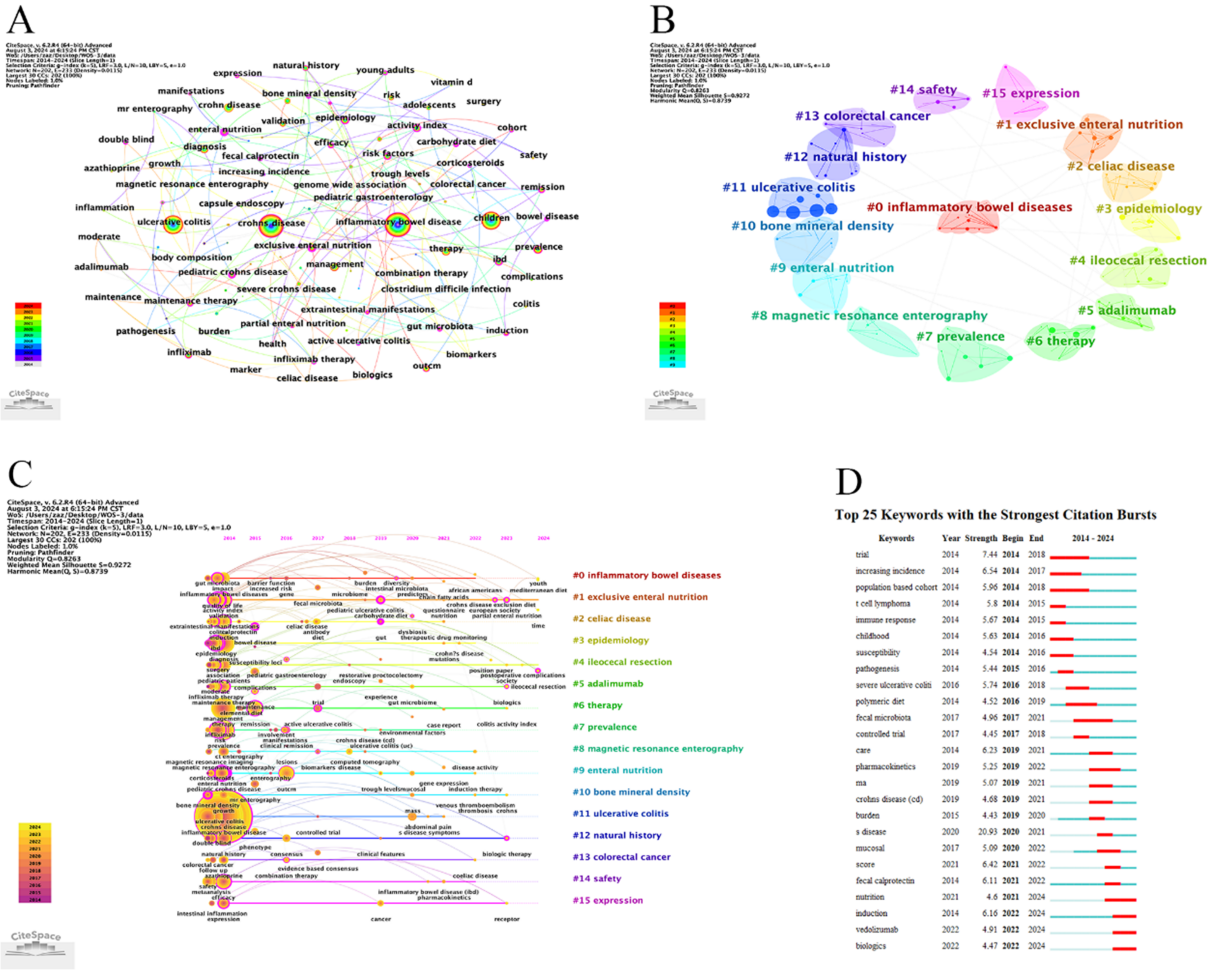
Analyse of keywords. **(A)** Visualization of keywords. **(B)** Clustering map of Keywords. **(C)** Keyword timeline diagram. **(D)** The top 25 Keywords with the Strongest Citation Bursts.

The keyword co-occurrence network helps us to understand the research hotspots and research trends in this field. Table 7 lists the 10 keywords with the highest frequency of occurrence during the 10-year period; combined with Figure 7A, it is easy to see that the highest frequency of occurrence is inflammatory bowel disease, followed by crohns disease and ulcerative colitis. among them, the highest centrality is ENTIRAL NUTRITION. It can be inferred that this is an important direction in the treatment of CD in children. The historical evolution of the keywords can be seen in Figure 7C. The research hotspots evolved from the pathogenesis of CD in children to the social impacts in the middle of the journey, and then to the new biologics nowadays. with the development of modern medicine, the diagnosis and treatment of the disease have formed a more complete system, but in recent years, the treatment methods are still changing, and in addition to the application of the biologics, we should also try to explore more effective treatment methods. Figure 7B Cluster analysis of keywords by applying Citespace, a total of 16 clusters were obtained. The focus is on epidemiology, etiology and treatment. As shown in Figure 7D, we analyzed the top 25 keywords in terms of outbreak intensity, focusing on diagnosis, treatment and psychology. Among them, biologic therapy for CD in children is the focus of extensive attention in recent years.

**Table 7:** Top 10 keywords with the highest frequency of occurrence.

Rank	Keywords	Occurrences	Keywords	Centrality
1	Inflammatory bowel disease	1,673	Enteral nutrition	0.6
2	Crohns disease	1,658	Exclusive enteral nutrition	0.59
3	Ulcerative colitis	1,270	Pediatric crohns disease	0.56
4	Children	1,171	Carbohydrate diet	0.55
5	Crohn disease	424	Bone mineral density	0.45.
6	Therapy	367	Bowel disease	0.44
7	Management	349	Gut	0.42
8	Diagnosis	309	Biologic therapy	0.35
9	Prevalence	249	Young adults	0.34
10	Validation	197	Maintenance therapy	0.33

## 4 Talk over

This study is the first to use CtieSpace and VOSviewer to visualize and analyze the literature in the field of pediatric Crohn's disease and to summarize the research frontiers and hotspots in the field. A total of 3,177 publications from January 1, 2014 to August 2, 2024 were collected for this study. The publication trend of the literature is an overall upward trend showing a steady increase, in which the publication of the literature in 2021 reached the highest number of 370 articles in the past 10 years, and since then there are fluctuations in the literature published every year, but still at a high level, indicating that the researchers' enthusiasm for research in this field is only increasing.

Turner, Dan is the first author with the highest number of publications and the second most cited author with more research on biomarkers of CD and treatment with biologics (metabolism and monoclonal antibodies) (12–17). The first author with the highest number of citations is Levine, A. Levine, A and his team showed that exclusion of dietary therapy with or without enteral nutrition may induce clinical remission in Crohn's disease (18, 19).

The United States and Canada are both highly productive countries in the field of pediatric CD. Because the disease is closely related to the dietary structure of developed countries, the number of cases in these two countries is high, which has prompted them to carry out profound research on the disease and produce quite fruitful results. Looking at the top ten countries in the publication, eight of them belong to European and American developed countries, and only two are from Asian countries, of which only China is a developing country. With the globalization of the disease, European and American developed countries should strengthen the cooperation and contact with Asian and African countries, so as to provide more comprehensive and cutting-edge diagnostic and treatment guidelines for children with CD. Among the top ten institutions, the sources show that they are also monopolized by the United States, Canada and Israel, with six institutions from the United States, 20% from Canada and 20% from the United States, and Tel Aviv University from Israel occupying the first place, which shows that this institution pays more attention to children with CD and contributes to the research in the field of children with CD. Combined with the literature, we know that North America and Europe have the highest prevalence rates, which are still increasing or stable, while the newly industrialized countries in Asia, the Middle East, and Africa are also experiencing an increase in prevalence rates (2). The incidence is also increasing in newly industrialized countries in Asia, the Middle East, and Africa. We have found that because developing countries have fewer cases than developed countries, developed countries have more experience and foresight in the study of this disease, but with the globalization of CD in children, global coordination should be encouraged, and developing countries should strengthen their cooperation with developed countries in order to promote the development of research in the field of CD in children, and to construct more authoritative and comprehensive guidelines, rather than geographically specific consensus and guidelines. The following is a summary of the recommendations of the study.

The top-ranked journal, Journal Of Pediatric Gastroenterology And Nutrition, IF 2.4 Q3, published 396 articles on pediatric CD. Only one of the top ten journals has an IF lower than 2, and six journals are in JCR division 1, indicating that the research on pediatric CD is a hot topic of global concern, the overall academic level is high, and the results of the research are authoritative and instructive.

The most cited literature is ESPGHAN Revised Porto Criteria for the Diagnosis of Inflammatory Bowel Disease in Children and Adolescents, an article that takes an evidence-based and consensus approach using a robust methodological approach to the Porto criteria was revised and a new approach to diagnosing IBD-unclassiﬁed (IBD-U) based on a multi-criteria approach was presented (20). The second most frequently cited article was Consensus guidelines of ECCO/ESPGHAN on the medical management of pediatric Crohn's disease, which was co-authored by ECCO/ESPGHAN using a benefit-risk analysis-based individualized treatment algorithm for Children and adolescents with Crohn's disease (CD) medication and long-term management to provide the most up-to-date state-of-the-art guidance. In children and adolescents with incomplete growth, exclusive enteral nutrition (EEN) is the treatment of choice for induction therapy, and the majority of patients with childhood-onset CD require maintenance therapy based on immunomodulators.This guideline only provides an approximate direction for treatment, but because there are so many different clinical scenarios, it should be individualized in treatment (21). The article with the highest intensity of outbreaks was The Medical Management of Paediatric Crohn's Disease: an ECCO-ESPGHAN Guideline Update, which provides a review of a 2014 article published in the Journal of Crohns &amp Colitis, which updates the consensus guidelines on Children and adolescents with CD published in 2014, with highlights of the update including: proposing new goals for the treatment of Crohn's disease in children; advocating for the optimization of individualized treatment regimens and proposing risk stratification; and developing different induction and maintenance treatment regimens for different risk strata of children (22). The update highlights the following.

## 5 Hot topics and frontiers

The frequency and centrality of keywords shows that the therapeutic aspect dominates pediatric CD research, with drug therapy, immunomodulators, corticosteroids, biologics, surgical therapy, other therapies (enteral nutrition), etc (3, 23). Under a variety of therapies, enteral nutrition therapy has become the center of attention.

Enteral nutrition includes total enteral nutrition (EEN) and partial enteral nutrition (PEN), with total enteral nutrition standing out amongst the various approaches because of its significant remission-inducing effect and the absence of virtually all medical side effects (21, 24, 25). It stands out among the various approaches as the first-line agent for inducing remission in active pediatric Crohn's disease, with typical remission rates of up to 85% (26). Studies have shown that EEN therapy can induce remission in children with active Crohn's disease. Studies have shown that EEN therapy induces mucosal healing, promotes growth and significantly improves nutritional parameters, improves bone mineral density, alters intestinal flora, enhances barrier function and has a direct anti-inflammatory effect (26, 27). Although EEN has better clinical remission, the method requires extreme family cooperation, which is challenging for the average family. Considering that the treatment of CD in children needs to be individualized, we should take into account multiple factors, such as age, site of disease, disease behavior, the presence of growth delays, potential side effects of medications, and quality of life. A key point in developing an optimal treatment plan is how to identify high-risk patients with a complex disease course, with the overall goal of rapid control of inflammation and reduction of long-term intestinal damage. It is therefore the responsibility of each clinician to adapt these guidelines to local regulations as well as to the individual characteristics and needs of the patient.

## 6 Strengths and limitations

No researcher has published a bibliometric analysis of pediatric CD research. This study summarizes comprehensive information and hot frontiers in CD from the perspective of bibliometric analysis using Citespace (6.1.R6) and VOSviewer (1.6.18) to provide a meaningful reference for research in this field. This study is not perfect and has limitations. First, there may be subjectivity in manually screening the included literature. Second, we retained only English literature. Finally, VOSviewer and CiteSpace do not support advanced statistical analysis, which may introduce statistical bias. However, these limitations do not affect the extraction and analysis of comprehensive information in the field of children's CD.

## 7 Reach a verdict

The bibliometric analysis of this study presents comprehensive information on articles in the field of pediatric CD over the last decade. We found that pediatric CD research is undergoing a rapid development phase, with more and more countries, institutions and researchers taking a keen interest in pediatric CD. Although results continue to emerge, the treatment of pediatric CD remains a pressing issue and a focus for future research. We summarize and propose innovations for global collaboration in pediatric CD. We hope that the “country-institution-journal” model of collaboration will help researchers and clinicians make more extraordinary breakthroughs in this field.
